# Crescentic “Semilunar” Cap‐Like Pus–Fluid Level in Bacterial‐Fungal Brain Abscess

**DOI:** 10.1002/ccr3.72821

**Published:** 2026-06-28

**Authors:** Nazmin Ahmed, Mohammad Nazrul Hossain, Bipin Chaurasia

**Affiliations:** ^1^ Department of Neurosurgery Ibrahim Cardiac Hospital & Research Institute Dhaka Bangladesh; ^2^ Department of Neurosurgery Neurosurgery Clinic Birgunj Madhesh Pradesh Nepal

**Keywords:** bacterial‐fungal, brain abscess, crescentic (semilunar) cap, pus–fluid level

## Abstract

A crescentic (“semilunar”) cap‐like pus–fluid level seen across CT/MRI sequences in a polymicrobial (bacterial‐fungal) brain abscess which may aid preoperative recognition when interpreted alongside clinical context and DWI/ADC.

**Abbreviations**
ADC: apparent diffusion coefficient
C/S: culture sensitivity
CT: computed tomography
DWI: diffusion‐weighted imaging
IV: intravenous
MRI: magnetic resonance imaging
T1WI: T1‐weighted image
T2WI: T2‐weighted image

## 1 Image Report

A 22‐year‐old man with a known history of intravenous drug abuse presented with a 2‐week history of headache, fever, and right‐sided weakness with two episodes of convulsion within this period. Physical examination revealed muscle power 3/5 on the right side, upper motor neuron type of facial palsy, and papilledema. Initial CT scan of the brain demonstrated multiple coalescing ringlike lesions in the parietal periventricular region, surrounded by moderate to severe vasogenic edema (Figure 1). The patient was prophylactically prescribed IV antibiotic outside our facility. However, rapid clinical and neurological deterioration warranted further evaluation with MRI (Figure 2), which demonstrated multiple, coalescing, rim enhancement, severe vasogenic edema, and 12 mm midline shifting. Instead of the classic “dual rim” sign, there was evidence of a well‐demarcated crescentic semilunar area, capping the abscess cavity. Besides this, lesions extended from the periventricular white matter to infiltrate the overlying dura matter. The patient underwent parieto‐occipital craniectomy, evacuation of pus, and removal of pyogenic membrane with overlying involved dura matter. He was discharged on the fifth postoperative day with improved neurological status (muscle power became 4/5). Microscopic examination features were consistent with granulomatous inflammation (Figure 3). Besides this, pus culture revealed growth of yeast with moderate hyphae and *Staphylococcus aureus* . Concomitant bacterial‐fungal brain abscess was diagnosed accordingly. The patient was prescribed linezolid with ciprofloxacin for 3 months and voriconazole for 6 months, according to the C/S report. At the 6‐month follow‐up, CT scan confirmed complete resolution of the lesion, mild extracalvarial herniation through craniectomy defect with encephalomalachic change (Figure 4). He then underwent cranioplasty with autologous bone flap and achieved complete neurological recovery.

**FIGURE 1:**
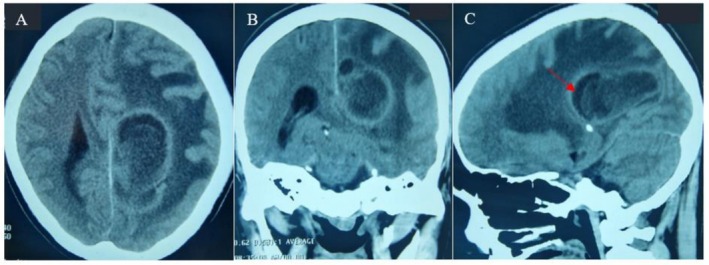
CT scan of brain, axial (A), coronal (B), and sagittal (C) sections demonstrates multiple intraparenchymal coalescing cystic lesions of varying sizes and shapes located in the paraventricular, parietal, and insular regions of the left cerebral hemisphere. These display marginal hyperdense signal change with heterogeneously hypodense content, surrounded by massive perilesional edema. A crescentic intrinsic hypodense area capping the lesion is noted (marked by red arrowhead).

**FIGURE 2:**
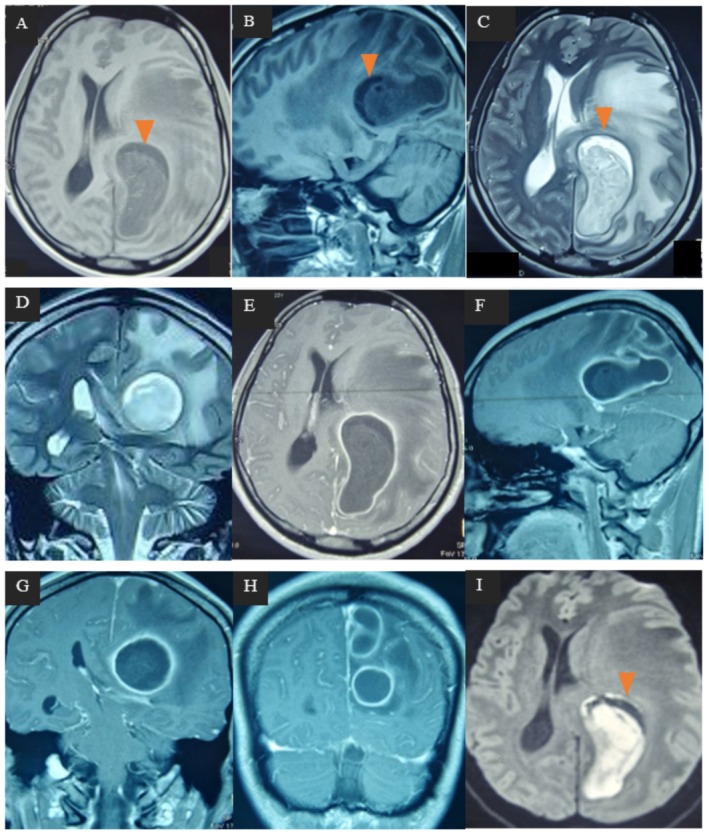
MRI of brain, T1WI axial (A) and sagittal (B) sections demonstrates a well‐delineated pear‐shaped iso‐ to hypointense lesion with an intrinsic dense hypointense capping area located in the left paraventricular region. The lesion becomes heterogeneously hyperintense on T2WI surrounded by a dense hypointense rim (C, D). The lesion shows multiple coalescing irregular rim enhancements after administration of gadolinium (E–H). On DWI/ADC, most of the content showed restricted diffusion skipping a semilunar area capping the largest abscess cavity (I). There is grade III edema that involves the whole cerebral hemisphere with significant mass effect upon the ipsilateral lateral ventricle with 15 mm midline shift. No dual rim sign is evident; rather, a new “capping sign” is visible in all sequences (marked by orange arrowhead).

**FIGURE 3:**
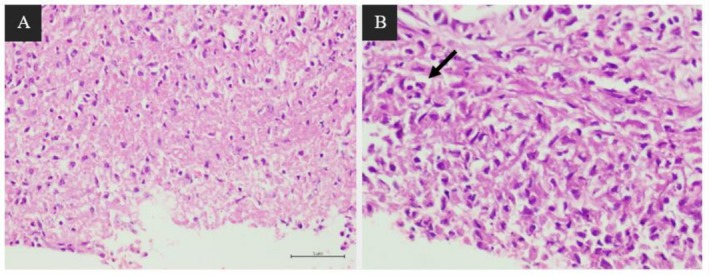
Photomicrograph of hematoxylin and eosin stain (10× magnification) shows part of an abscess wall composed of fibrinopurulent exudate resting on granulation tissue (A). At the edge of suppuration, collection of palisaded epithelioid histiocytes are seen (marked by black arrow in 20× magnification). No caseous necrotic material present (B).

**FIGURE 4:**
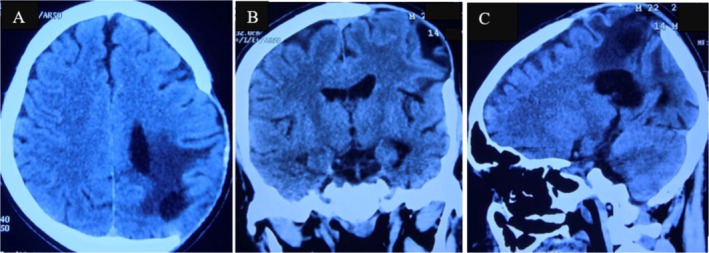
Follow‐up CT scan (A–C) after completion of 6 months of antifungal therapy shows compete resolution of the lesion. There are some encephalomalachic changes with mild extracalvarial herniation.

## 2 Discussion

Brain abscess is considered a serious complication in IV drug abusers with natural commensal organism [1]. Concomitant presence of bacterial‐fungal brain abscess remains a diagnostic challenge due to misleading radiological features. Diagnostic radiological signs for bacterial or fungal brain abscess were absent in our reported case. Instead of classic location in gray‐white junction, the abscess cavity predominantly involved periventricular white matter, spared basal ganglia, and extended to infiltrate the dura mater [1, 2]. Besides these atypical radiological presentations, we observed a well‐defined crescentic area, capping the abscess cavity in all sequences of MRI, which we defined as a “semilunar sign” in this complex brain abscess case. According to the previous literature, fluid levels can be detected in different pathologies of the brain. Morris and his colleagues prospectively reviewed 17 intracranial pathologies, having blood fluid level; all of them were neoplastic and vascular in origin [3]. Besides this, gas fluid level can be detected in brain abscess, whereas fat fluid level can be a rare imaging feature of intracranial dermoid cysts [3]. It is hypothesized that disruption of the blood–brain barrier, necrobiosis of the tumor cells, and gas‐producing organisms are responsible for the imaging appearance of the fluid level. In our reported case, we hypothesized that due to bacterial‐fungal symbiosis, abscess produced by the respective organism was believed to be different in both consistency and composition, which manifested as this “semilunar sign” in the imaging which may play a pivotal role in preoperative diagnosis, warranting prompt surgical exploration.

### 2.1 Limitation

This is a single‐case observation and the semilunar cap morphology is not specific. Moreover, there is an absence of archived microbiology images.

## 3 Conclusion

A crescentic (“semilunar”) cap‐like pus–fluid level can occur in polymicrobial brain abscess. Recognizing this radiological sign will expedite appropriate surgical and antimicrobial management.

## Author Contributions

**Nazmin Ahmed:** conceptualization, data curation, formal analysis, writing – original draft. **Mohammad Nazrul Hossain:** validation, visualization. **Bipin Chaurasia:** supervision, validation, visualization, writing – review and editing.

## Funding

The authors have nothing to report.

## Consent

Written informed consent was taken from the patient prior to the initiation of this project.

## Data Availability

Data sharing not applicable as no new data were generated. The article describes entirely theoretical research.
